# Fibre-Reinforced Polymer Composites: Mechanical Properties and Applications

**DOI:** 10.3390/polym14183732

**Published:** 2022-09-07

**Authors:** R. A. Ilyas, S. M. Sapuan, Emin Bayraktar, Shukur Abu Hassan, Nabil Hayeemasae, M. S. N. Atikah, Khubab Shaker

**Affiliations:** 1 School of Chemical and Energy Engineering, Faculty of Engineering, University Teknologi Malaysia (UTM), Johor Bahru 81310, Malaysia; 2 Centre for Advanced Composite Materials, University Teknologi Malaysia (UTM), Johor Bahru 81310, Malaysia; 3 Institute of Tropical Forest and Forest Products (INTROP), University Putra Malaysia (UPM), Serdang 43400, Malaysia; 4 Advanced Engineering Materials and Composites, Department of Mechanical and Manufacturing Engineering, Faculty of Engineering, University Putra Malaysia (UPM), Serdang 43400, Malaysia; 5 School of Mechanical and Manufacturing Engineering, ISAE-SUPMECA Institute of Mechanics of Paris, 93400 Saint-Ouen, France; 6 Department of Rubber Technology and Polymer Science, Faculty of Science and Technology, Prince of Songkla University, Pattani Campus, Pattani 94000, Thailand; 7 Department of Chemical and Environmental Engineering, Faculty of Engineering, University Putra Malaysia (UPM), Serdang 43400, Malaysia; 8 Department of Materials, School of Engineering and Technology, National Textile University, Punjab 37610, Pakistan

*"Fibre-Reinforced Polymer Composites: Mechanical Properties and Applications"* is a newly open Special Issue of *Polymers*, which aims to publish original and review papers on new scientific and applied research and make boundless contributions to the finding and understanding of the reinforcing effects of various synthetic and natural fibres on the performance of biopolymer composites. This Special Issue also covers the fundamentals, characterisations, and applications of synthetic and natural fibre-reinforced biopolymer composites.

Rapid growth in the manufacturing industries has necessitated the improvement in materials in terms of density, stiffness, strength, and cost-effectiveness with increased sustainability. Composite materials have been developed as one of the materials with such improvements in these qualities that serve their promise in various applications. Composite materials are made up of two or more elements, one of which is present in the matrix phase (synthetic or biopolymer [1,2,3,4,5,6]) and the other in particle or fibre form. Composites have been discovered to be the most promising material available in the twenty-first century. Composites reinforced with synthetic or natural fibres are becoming extremely prevalent as the market grows in demand for lightweight materials with high strength for specific applications. The matrix, which serves primarily to hold the reinforcement together, is also regarded as resin, particularly in the case of polymers.

In order to create eco-friendly composites, natural fillers such as natural fibres [7,8,9], nanocrystalline cellulose, nanofibrillated cellulose [10,11], bacterial nanocellulose [12], and chitosan have been added to the polymer matrix. This increased material qualities while minimising the problem of residue formation. Many researchers have reported the benefits of cellulosic fibres, including the fact that they are abundant in nature, renewable, cost-effective, and non-toxic, as well as providing necessary bonding with the cement-based matrix for significant improvements in material properties such as flexural capacity, toughness, ductility, and impact resistance.

Several studies that investigated the fibre-loading effect on polymer composites found that it had a good relationship with tensile strength. Studies on the fibre loading effect that led to the tensile strength were observed [13,14]. It was demonstrated that the optimum fibre loading for kenaf/thermoplastic polyurethane composites was 30% [15]. Other studies regarding kenaf fibre and phenol-formaldehyde (KF/PF) composites reported that kenaf fibre loading up to 43% showed the best tensile strength for the composites [16].

A study on a kenaf-fibre-reinforced, corn-starch-based biocomposite film investigated the fibre loading effect for the tensile properties. It was found that at 6% of the kenaf fibre loading, the optimum tensile properties (17.74 MPa) were observed [17]. Studies on *Cymbopogan citratus* fibre with cassava starch showed higher tensile properties (19.27 MPa) with the use of 50% fibre loading [18]. Another study on arrowroot (*Maranta arundinacea*)-fibre-reinforced arrowroot starch biopolymer composites conducted by Tarique et al. [19] shows that the mechanical properties were enhanced up to 15.22 MPa with the optimum filler content was 10%. Previous studies on the correlation of fibre length to tensile strength were conducted by several researchers. Studies on wheat husk length reinforced rubber composites showed that the highest tensile strength achieved was the medium length of fibre (125–250μm), where the fibre was arranged longitudinally [20]. Jihua Zhu [21] conducted a study on glass-fibre-reinforced composites under acid–base and salt environments. The results indicated that the tensile strength of the GFRP decreased by 22%, 71%, and 87% after 56 d of exposure to 5% NaOH solutions at 20 °C, 50 °C, and 80 °C, respectively.

Not only do fibre-reinforced polymer composites have a high strength-to-weight ratio, but they also have excellent properties such as high durability, stiffness, damping property, flexural strength, and resistance to corrosion, wear, impact, and fire. Various properties of composites materials have led to applications in construction, aerospace, packaging [22,23,24], electronic, electrical, structural, energy storage [25], automotive [26], filter, coating, bone tissue engineering, and drug delivery [27] and many other industries. Because the performance of composite materials is primarily determined by their constituent elements and manufacturing techniques, the functional properties of various fibres available worldwide, their classifications, and the manufacturing techniques used to fabricate the composite materials must be investigated.

This Special Issue will also cover recent advances in composite processing, mechanical characterisation, and potential applications. Both synthetic and natural fibre-reinforced polymer composites are welcome. Moreover, we welcome approaches to this issue from several vital directions, such as the production of fibres, surface and interfacial characterisation of its properties, economic feasibility, challenges, and future perspectives in the field of polymer composites. As a result of this Special Issue, current and future literature data can be enriched.

## Data Availability

Not applicable.
